# Interaction between trait and housing condition produces differential decision-making toward risk choice in a rat gambling task

**DOI:** 10.1038/s41598-017-06408-4

**Published:** 2017-07-18

**Authors:** Wha Young Kim, Bo Ram Cho, Myung Ji Kwak, Jeong-Hoon Kim

**Affiliations:** Department of Physiology, Brain Korea 21 Project for Medical Science, Brain Research Institute, Yonsei University College of Medicine, Seoul, 03722 South Korea

## Abstract

Poor decision-making is a core problem in psychiatric disorders such as pathological gambling and substance abuse. Both trait and environmental factors are considerably important to affect decision-making. However, it has not yet been systematically shown how they interact to affect risk preference in animal models evaluating decision-making. Here, we trained rats, housed in pairs or in isolation, in a touch screen chamber to detect the association between four different light signals on the screen and accompanied reward and punishment outcomes arranged with different schedules. Then, the rats were allowed to freely choose from 4 different light signals. Once animals showed a stabilized pattern of preference (risk-averse or risk-seeking), they were injected with saline or cocaine (a single injection per day for 7 days) followed by 2 weeks of withdrawal. Then, their preference of choice was re-tested in the touch screen chamber while they were cocaine challenged. All rats significantly changed their preference toward more risky choices when they were exposed to and challenged with cocaine, except those in the risk-averse/isolated housing group. These results indicate that the pre-existing trait toward risk and the housing condition interact to affect the quality of decision-making, and cocaine may help to aggravate this process.

## Introduction

It is important to make proper and better decisions in our daily life to maximize long-term benefits. Poor decision-making will eventually have harmful effects on an individual’s life, as demonstrated in people with some psychiatric conditions, such as pathological gambling and drug abuse^1–3^. Adopting the basic principle of the Iowa Gambling Task (IGT), which is widely used to assess decision-making in humans, the rodent version of the gambling task (rGT) has been developed in a few laboratories^4, 5^. Importantly, the rGT shares many of the features of the human gambling tasks, including uncertainty, reward, and punishment; which makes translational research possible with an animal model of psychiatric disorders related to decision-making deficits, including behavioral addictions^4, 6^. Similarly, in the present study, we adopted one of the previously developed rGT models^7^, and trained the rats in a touch screen chamber^8^ to learn the associations between four different light signals on the screen and the accompanied reward or punishment outcomes set up with different schedules.

Environmental factors affect decision-making significantly. For example, human studies have shown that environmental factors such as finance, education, and employment selectively affect the co-occurrence of pathological gambling with specific drug-use disorders in young adult twins, while economic environmental factors affect risk aversion in financial decision-making in young students^9, 10^. Different from humans, however, environmental factors to consider in animals are more restricted and an easy way to manipulate them in the laboratory is to differentiate housing conditions^11–13^. With regard to rGT, to our knowledge, only a single study has thus far shown that different housing conditions (e.g., social vs. isolation) differentially affect this type of decision-making behavior^13^. While these results certainly indicate that housing conditions are considerable factors that affect decision-making, it is not determined yet whether sub-populations of animals, possibly showing differential preference toward risk, exist even among the animals raised in the same housing conditions, and if so, how they differentially respond to external stimuli; for example, to drugs of abuse via interactions between pre-existing preference toward risk and different housing conditions.

There is ample evidence that maladaptive decision-making behaviors are associated with an increase of cocaine use in both humans and animals^14–19^. It is also well known that cocaine, when repeatedly administered, produces sensitization to locomotor activity (psychomotor sensitization) as well as to approaching behavior toward conditioned or predictive stimuli paired with reward (incentive sensitization), which has been proposed to model the escalation of drug use and craving that is characteristic of drug addiction in humans^20–23^. However, with regard to rGT, it has not been assessed yet what effects cocaine, especially within the context of the sensitization-producing scheme, may have on risk-choice behavior.

To address these issues, in the present study, we examined how cocaine sensitization affects the preference scores in rGT according to the interactions between pre-existing trait toward risk and housing conditions.

## Results

### Effect of different housing conditions on basal scores for risk preference

**Figure 1:**
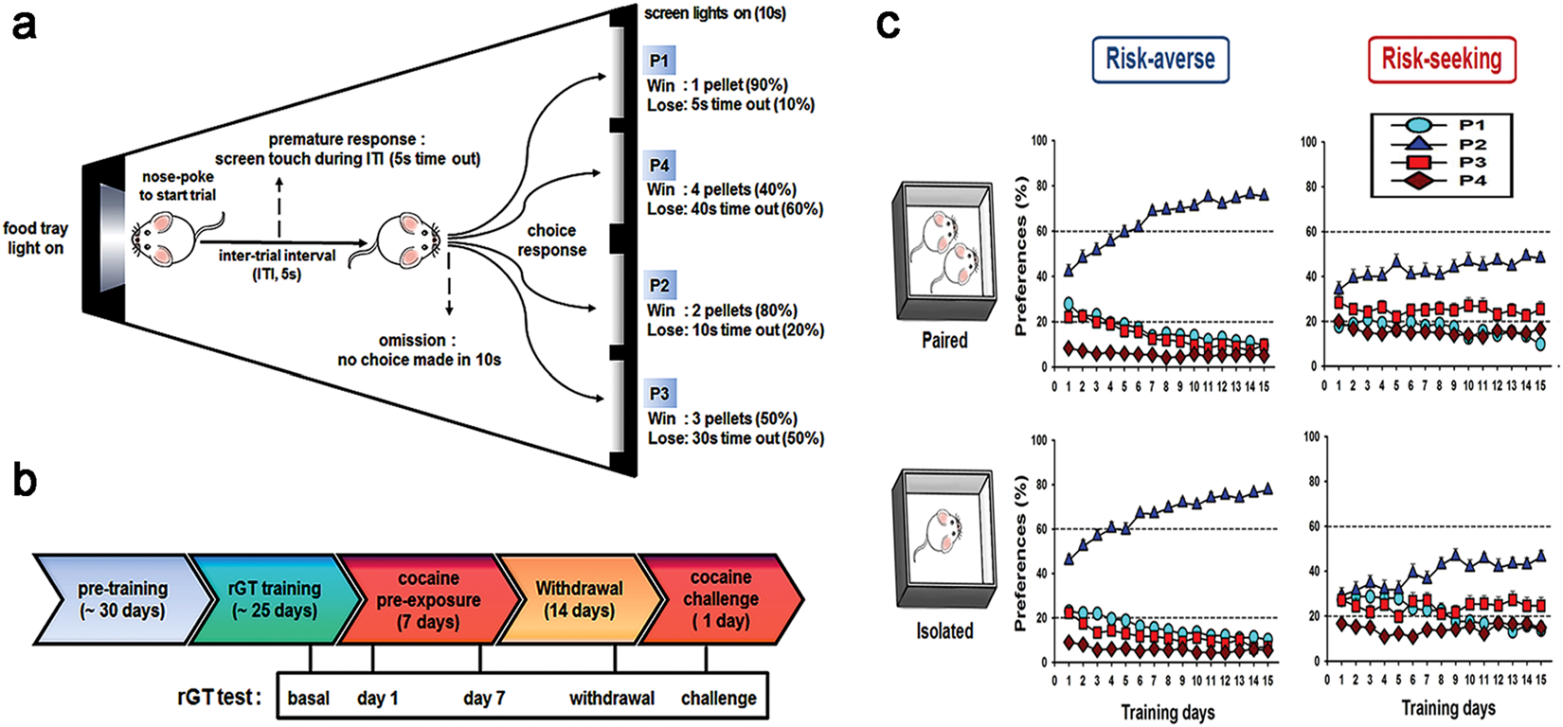
Rat gambling task apparatus, experimental schemes, and basal preference scores. (**a**) Schematic diagram of the rGT chamber, where a food magazine unit (left) and 4 response windows (right) are shown. Each window is represented as P1 through P4 with a different number of pellets, duration of time-out, and frequencies. (**b**) Time lines for the experimental procedures when the rGT tests were conducted are indicated. (**c**) Data obtained during the 15 consecutive days of stage 6 rGT training clearly show different preferences of choice between the groups. The risk-averse group overwhelmingly and consistently chose P2 over the other windows more than 60% of the choices after 6 days, whereas the risk-seeking group chose P2 less than 60% of the choices throughout the training days. The choice of P3 in the risk-averse group never increased more than 20% after 5 days of training, whereas it was always higher than 20% in the risk-seeking group throughout all the training days. There were no significant differences between the paired and isolated groups. However, there were small differences such as the choice of P3 decreased less than 20% faster in the isolated group than in the paired group (risk-averse), the choice of P1 decreased less than 20% later, and the choice of P2 was relatively a little lower in the isolated group than in the paired group (risk-seeking). The numbers of rats in each group were as follows: averse-paired (16), averse-isolated (20), seeking-paired (14), and seeking-isolated (16).

**Figure 2:**
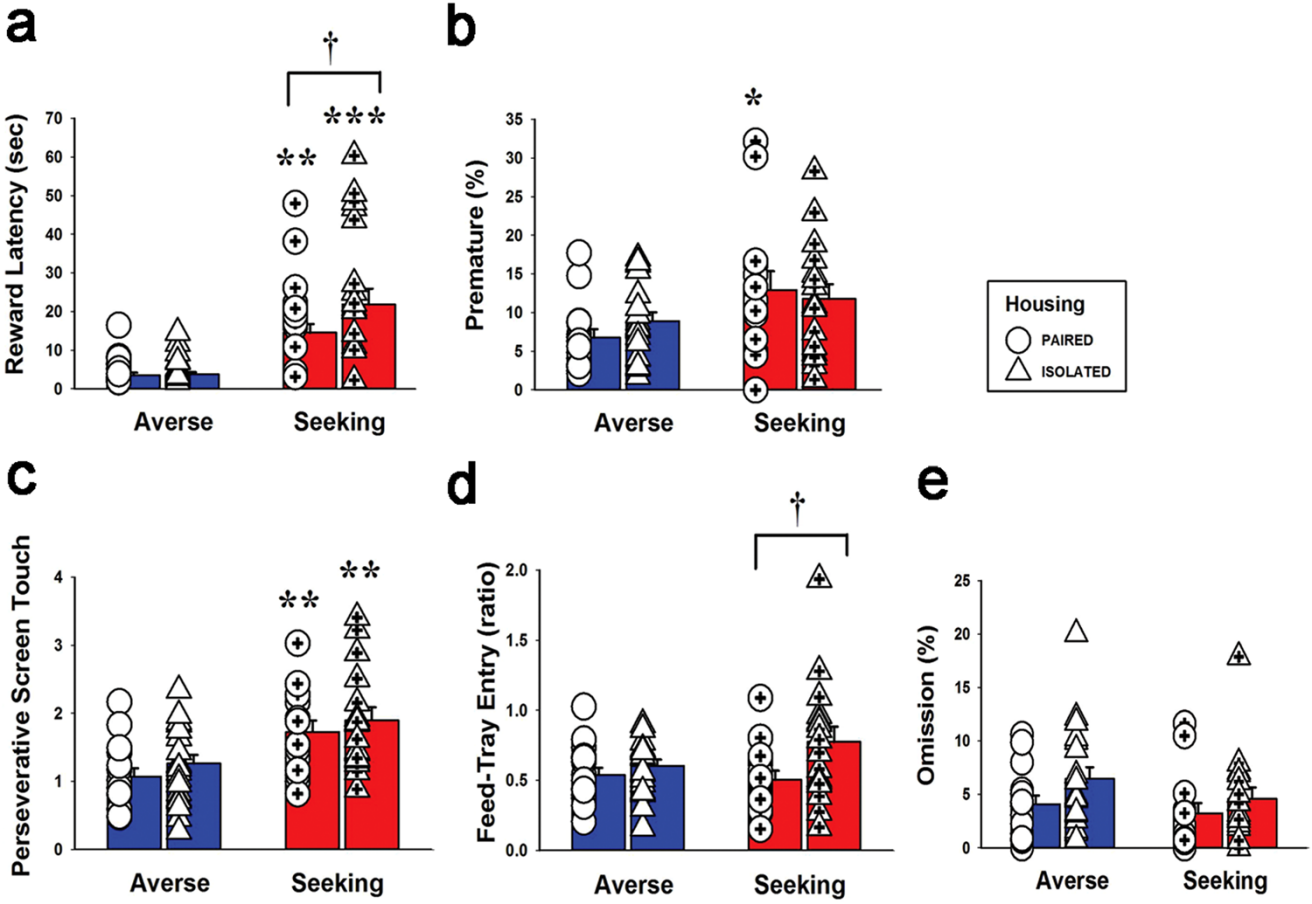
Analysis of behavioral parameters for the data obtained during the basal stage. The numbers of rats in each group were as shown in Fig. 1c. Values are expressed as mean + standard error of the mean. (**a**) Reward latency. **p < 0.01; significantly higher in the seeking-paired than in the averse-paired group. ***p < 0.001; significantly higher in the seeking-isolated than in the averse-isolated group. ^†^ *p* < 0.05; significant differences between the seeking-paired and seeking-isolated groups. (**b**) Premature response. *p < 0.05; significantly higher in the seeking-paired compared to the averse-paired group. (**c**) Perseverative screen touch. **p < 0.01; significantly higher in the seeking-paired compared to the averse-paired group. **p < 0.01; significantly higher in the seeking-isolated compared to the averse-isolated group. (**d**) Feed-tray entry. ^†^ *p* < 0.05; significant differences between the seeking-paired and seeking-isolated groups. (**e**) Omission. There was no significant difference between groups.

When allowed to freely choose light signals from the four different windows in the rGT, rats were clearly divided into risk-averse and risk-seeking groups, according to their stabilized preference for advantageous (P1 or P2) or disadvantageous choices (P3 or P4) (Fig. 1c **)**. There were no differences in terms of risk preference at the basal level between the paired and isolated rats. However, when we more closely analyzed the several choice-related behavioral parameters, it was found that the risk-seeking group had higher scores than the risk-averse group in reward latency, premature response, and perseverative screen touch (Fig. 2a–c). The results of the two-way ANOVA conducted on these data showed a significant effect of trait (averse vs. seeking) (F_1,62_ = 41.5, *p* < 0.001 for reward latency; F_1,62_ = 7.7, *p* = 0.007 for premature response; F_1,62_ = 17.9, *p* < 0.001 for perseverative screen touch). Interestingly, within the same risk-seeking group, isolated housing contributed to significantly increased scores for reward latency and feed-tray entries compared to paired housing (Fig. 2a,d). Post hoc Bonferroni comparisons revealed that the seeking-isolated groups had significantly higher scores in reward latency (*p* = 0.032) and feed-tray entries (*p* = 0.011) than seeking-paired groups. The omission score did not differ between any groups (Fig. 2e).

### Chronic cocaine administration differentially modifies risk preference depending on the interaction of trait and housing condition

**Figure 3:**
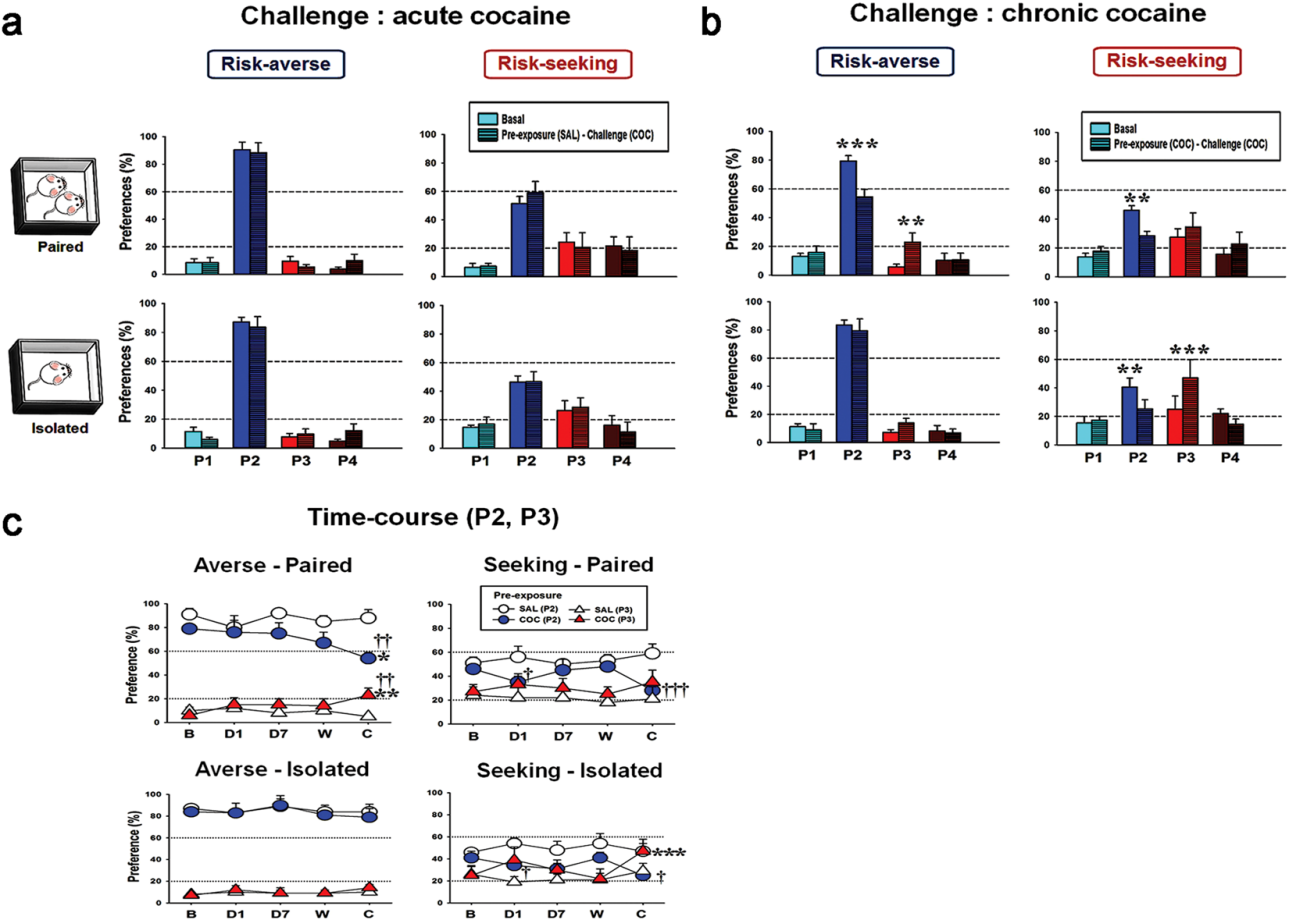
Preference scores after cocaine challenge. (**a**) The cocaine challenge in saline pre-exposed rats did not make any differences in the risk preferences compared to their basal scores. The numbers of rats in each group were as follows: averse-paired (7), averse-isolated (10), seeking-paired (6), and seeking-isolated (8). (**b**) In response to the cocaine challenge, when they were cocaine pre-exposed, all rats, except those in the averse-isolated group, had a more risk-seeking choice preference (i.e., they chose P2 less and P3 more). The numbers of rats in each group were as follows: averse-paired (7), averse-isolated (7), seeking-paired (8), and seeking-isolated (6). Values are expressed as a mean + standard error of mean. (**c**) In the saline and cocaine pre-exposed groups, the preference scores for only P2 and P3 obtained during the tests conducted at basal (B), pre-exposure day 1 (D1), pre-exposure day 7 (D7), the withdrawal period (W), and challenge day (C) are depicted. Again, it is clear that all rats, except those in the averse-isolated group, had more risk-seeking choice preference (i.e., they chose P2 less and P3 more) in response to the cocaine challenge, when they were pre-exposed to cocaine. Two-way repeated-measures ANOVA was conducted on data for P2 and P3 separately, but they were drawn together on the same graph for easy comparison.

Next, we examined the rGT preference scores upon cocaine challenge after 2 weeks of withdrawal following saline or cocaine pre-exposure. All rats with cocaine pre-exposure, except those in the averse-isolated group, showed preference changes toward risk-seeking choices when they were cocaine challenged, whereas there were no such effects observed in rats with saline pre-exposure (Fig. 3a,b). In the averse-paired group, results of the two-way repeated-measures ANOVA conducted on these data showed significant effects of windows (F_3,6_ = 40.3, *p* < 0.001), drug state (F_1,6_ = 11.5, *p* = 0.015), and windows × drug state interaction (F_3,6_ = 10.0, *p* < 0.001). Post hoc Bonferroni comparisons revealed that the rats with cocaine pre-exposure that were cocaine challenged chose P2 significantly less often (*p* < 0.001), and P3 significantly more often (*p* = 0.002) compared to their basal scores. In the seeking-paired group, there was only a significant effect on the windows × drug state interaction (F_3,7_ = 4.1, *p* = 0.02). Post hoc Bonferroni comparisons revealed that the rats with cocaine pre-exposure that were cocaine challenged chose P2 significantly less often (*p* = 0.002) compared to their basal score. In the seeking-isolated group, there was only a significant effect on the windows × drug state interaction (F_3,5_ = 7.5, *p* = 0.003). Post hoc Bonferroni comparisons revealed that the rats with cocaine pre-exposure that were cocaine challenged chose P2 significantly less often (*p* = 0.009), and P3 significantly more often (*p* < 0.001) compared to their basal scores. These differential effects between the groups on preference change upon cocaine challenge were further evident by examining the time-course data for rGT obtained from different time spots (basal, cocaine injection days 1 and 7, withdrawal, and challenge) (Fig. 3c). In the averse-paired group, there were significant effects of pre-exposure (for P2) (F_1,12_ = 5.4, *p* = 0.039) and pre-exposure × time interaction (for P3) (F_4,48_ = 2.7, *p* = 0.043). Post hoc Bonferroni comparisons revealed that the rats with cocaine pre-exposure chose P2 significantly less often (*p* = 0.016) compared to their basal score and less often (*p* = 0.002) than the rats with saline pre-exposure on the challenge day, whereas they chose P3 significantly more often (*p* = 0.005) compared to their basal score and the saline pre-exposed rats on the challenge day. In the seeking-paired group, there was only a significant effect of pre-exposure (for P2) (F_1,12_ = 8.5, *p* = 0.013). Post hoc Bonferroni comparisons revealed that the rats with cocaine pre-exposure chose P2 significantly less often (*p* < 0.001) than did the saline pre-exposed rats on the challenge day. In the seeking-isolated group, there were significant effects of pre-exposure (for P2) (F_1,12_ = 5.2, *p* = 0.041), time (for P3) (F_4,48_ = 6.8, *p* < 0.001), and pre-exposure × time interaction (for P3) (F_4,48_ = 4.3, *p* = 0.005). Post hoc Bonferroni comparisons revealed that the rats with cocaine pre-exposure chose P2 significantly less often (*p* = 0.018) than did the saline pre-exposed rats on the challenge day, whereas they chose P3 significantly more often (*p* < 0.001) compared to their basal score. Interestingly, in both seeking groups, rats with cocaine pre-exposure also chose P2 significantly less often (*p* = 0.013 or 0.029) than did saline pre-exposed rats on pre-exposure day 1.

### Analysis of Behavioral Parameters

**Figure 4:**
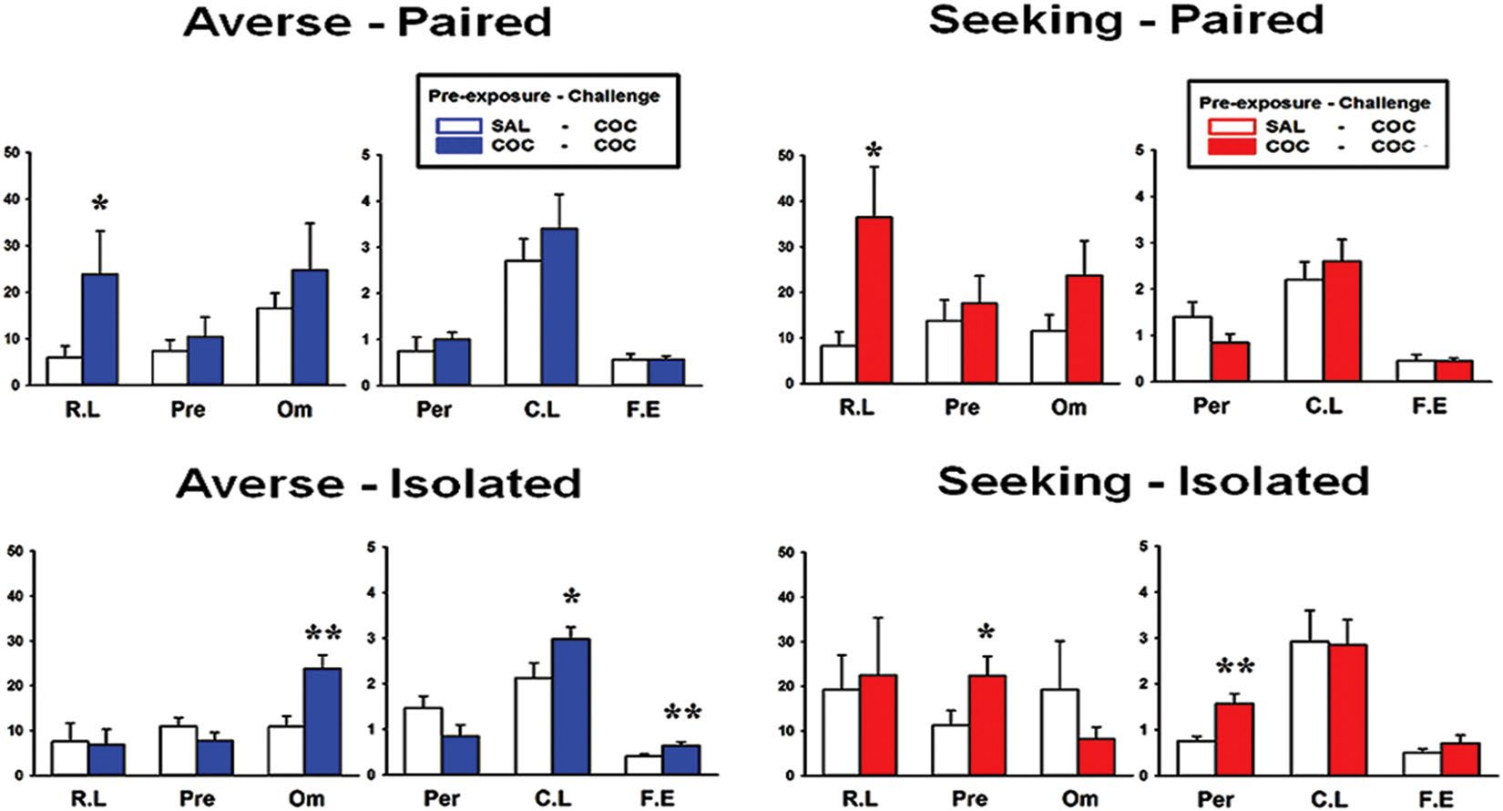
Analysis of behavioral parameters. The list of the choice-related behavioral parameters is as follows: reward latency (R.L), premature response (Pre), omission (Om), perseverative response (Per), correct response latency (C.L), and feed-tray entry (F.E). Each parameter’s units of measurement are as follows: R.L. (sec), Pre (%), Om (%), Per (actual number of touch), C.L. (sec), F.E (the number of feed-tray entries during ITI divided by total number of trials initiated). Data for these parameters obtained on the challenge day were analyzed using the independent sample t-test and compared between the saline and cocaine pre-exposed rats. In the averse-paired and seeking-paired groups, only differences for reward latency were significant between the saline and cocaine pre-exposed sub-groups. In the seeking-isolated group, differences in the premature and perseverative responses were significant between the saline and cocaine pre-exposed sub-groups. Interestingly, in the averse-isolated group, which did not show any change in choice preference upon the cocaine challenge, differences for the parameters such as omission, correct response latency, and feed-tray entries were significant between the saline and cocaine pre-exposed sub-groups.

**Figure 5:**
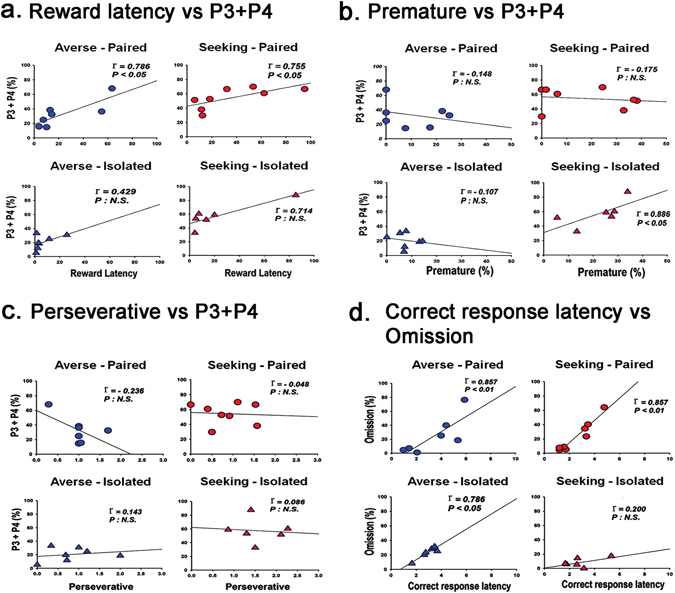
Correlation analysis of behavioral parameters. Correlation was analyzed using the Spearman rank-order on data obtained from cocaine pre-exposed and cocaine challenged rats. (**a**) A significant correlation was observed between reward latency and the P3 + P4 combined score (i.e., the disadvantageous choice) in the averse-paired and seeking-paired group. (**b**) There was only a significant correlation between premature responses and the P3 + P4 combined score in the seeking-isolated group. (**c**) There was no significant correlation between the perseverative response and P3 + P4 combined score in any group. (**d**) There was a significant correlation between the correct response latency and omission in all the groups, except the seeking-isolated group.

We further analyzed several choice-related behavioral parameters behavior^7, 24^ from the data obtained during the cocaine challenge. Remarkably, only reward latency was significantly higher in the cocaine compared to the saline pre-exposed sub-groups in the averse-paired and seeking-paired groups (t_12_ = 1.87, *p* = 0.043; t_12_ = 2.14, *p* = 0.027, respectively) (see Fig. 4, upper panels). Correlation analysis for the data obtained from the rats with cocaine pre-exposure that were cocaine challenged indicated that there were significant positive correlations between reward latency and disadvantageous choices (P3 + P4) only in the averse-paired and seeking-paired groups (*r* = 0.79, *p* = 0.025; *r* = 0.76, *p* = 0.021, respectively) (Fig. 5a). Interestingly, in the seeking-isolated groups, the premature and the perseverative responses were higher in the cocaine than the saline pre-exposed sub-groups (t_12_ = 2.08, *p* = 0.03; t_12_ = 0.81, *p* = 0.002, respectively) (see Fig. 4, right lower panel). Again, correlation analysis for these data indicated that there were significant positive correlations for the premature response with disadvantageous choices (P3 + P4) only in this group (*r* = 0.89, *p* = 0.033) (Fig. 5b), but these effects were not apparent in the perseverative response (Fig. 5c). The averse-isolated group, which did not show any change toward risk-seeking behavior, nonetheless showed changes in some minor behavioral parameters such as omission, correct response latency, and feed-tray entries with significant increases in cocaine compared to the saline pre-exposed sub-groups (t_15_ = 3.44, *p* = 0.002; t_15_ = 1.88, *p* = 0.04; t_15_ = 2.84, *p* = 0.006, respectively) (Fig. 4, left lower panel). The results of the correlation analysis indicated that there were positive significant correlations between omission and correct response latency in all groups, except the seeking-isolated group (Fig. 5d).

### Differential Expression of Locomotor Sensitization

**Figure 6:**
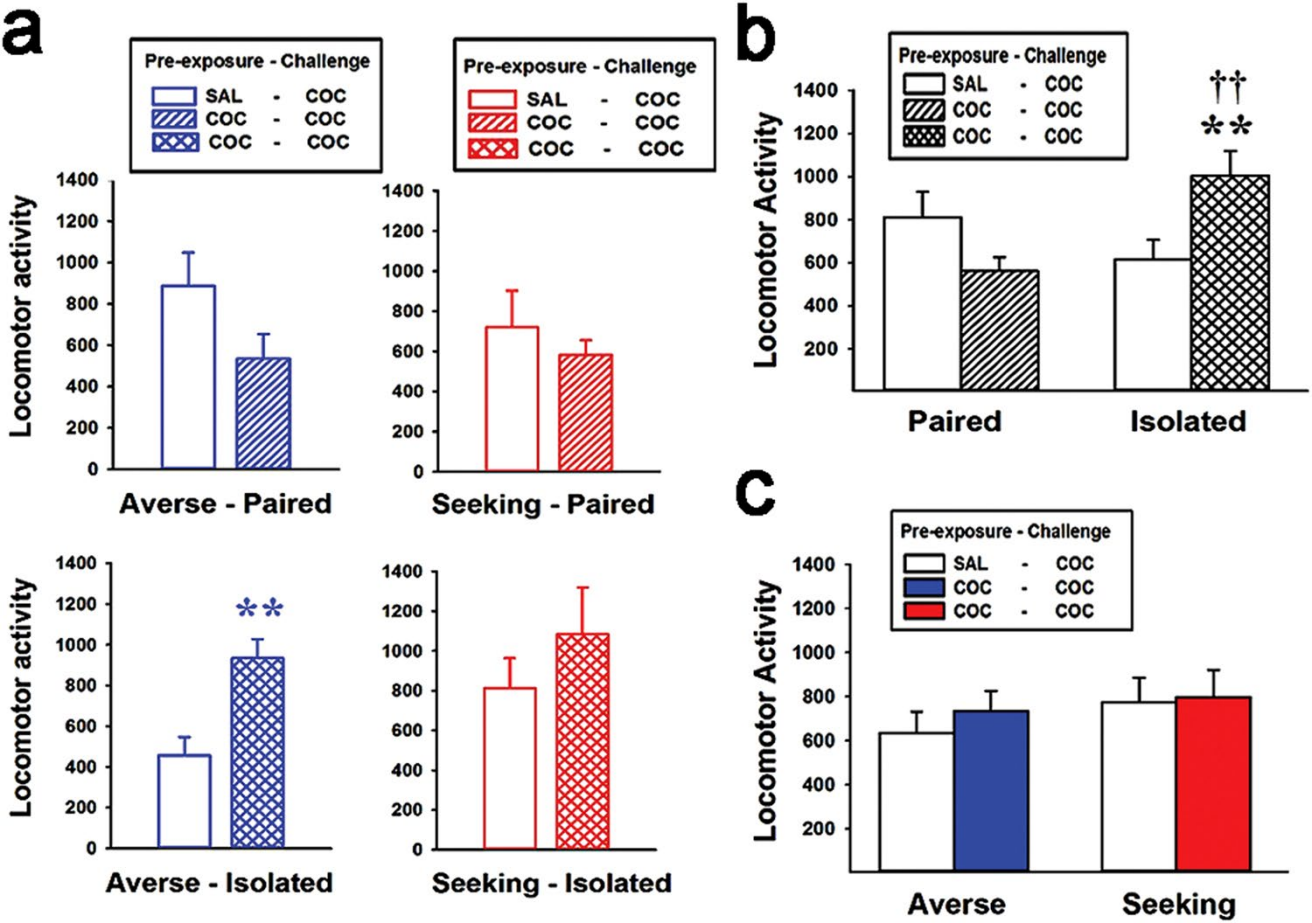
Locomotor activity. (**a**) During the cocaine challenge, locomotor activity (combined score of the horizontal locomotion and rearing) was significantly higher (i.e., locomotor sensitization) in cocaine compared to the saline pre-exposed sub-groups, but only in the averse-isolated group. (**b**) When the groups were combined and compared according to the housing environment (i.e., paired vs. isolated), locomotor sensitization only occurred in the isolated groups. (**c**) There was no significant difference between the averse and seeking groups when combined.

The procedure we used induces locomotor sensitization when cocaine is challenged after withdrawal^22, 25, 26^. As expected, we found that the cocaine challenge produced sensitized locomotor activity in cocaine compared to saline pre-exposed sub-groups; however, this observation only occurred in the averse-isolated group (t_15_ = 3.58, *p* = 0.001) (Fig. 6a). Interestingly, when we combined and re-analyzed data for the paired versus isolated housing groups, we found that only the isolated group displayed locomotor sensitization (Fig. 6b). The results of the two-way ANOVA conducted on these data showed a significant effect of environment × pre-exposure interaction (F_1,55_ = 10.5, *p* = 0.002). Post hoc Bonferroni comparisons revealed that there were significantly more counts in cocaine compared to saline pre-exposed animals in the isolated group (*p* = 0.006), while there were significant differences in cocaine pre-exposed animals between the paired and isolated groups (*p* = 0.003). These effects were not observed when we combined data into the averse and seeking groups (Fig. 6c).

## Discussion

The present results clearly showed that rats reared in the same housing conditions may be grouped according to their inclination toward risk preference. Further, our results revealed that, although rats in different housing environment (i.e., paired vs. isolated) have no difference in the basal preference scores in rGT within the same trait group categorized, they differentially change their preference scores when exposed to the sensitizing scheme of cocaine and challenged upon it. This is the first direct demonstration, to our knowledge, that chronic cocaine’s aggravating effects toward risky choice on decision-making can be differentially manifested depending on the interaction between pre-existing inclination toward risk-taking and housing environment.

It has been previously shown that isolation-reared rats did not make optimal choices compared to pair-housed rats in their basal performance in the rGT^13^. On the contrary, we showed, in the present study, that even isolation-reared rats were able to make optimal choices like pair-housed rats in their basal performance of the rGT (Fig. 1c). One major difference is that they reared rats in different housing condition starting at the very early age right after weaning (i.e., postnatal day 22), which is equivalent to the pre-pubertal period, before entering early adolescence, while we did at a later age (i.e., postnatal day 49), equivalent to mid to late adolescence in humans^27^. Considering the vulnerability that early-life adverse events bring to the development of the brain^28, 29^, behavioral traits developmentally influenced by the environment could be more strongly altered in rats reared in isolation starting earlier. However, in our present results, as we reared rats in different housing conditions starting later, after passing through the weaning and periadolescent periods, both pair-housed and isolation-housed rats clearly showed two distinct traits toward risk choice (i.e., averse and seeking), when allowed to freely choose in the rGT (Fig. 1c). Additionally, even considering their starting age in other experiments, there might be some variation existing among the rats reared in isolation showing higher scores toward optimal choices; however, the separation of such sub-groups was not shown and cannot be further compared. One additional difference is that we used a touch screen chamber, while others used conventional standard five-hole operant chambers, which might contribute to produce different ratios of sub-groups, although its effect could be much lower than the effect of the age differences in this case.

Although rats were pre-categorized according to their basal preference to risk-taking in each housing condition in our present results, these traits were not fixed and were rather modifiable by external stimuli, such as chronic cocaine administration. The group with a risk-averse inclination at the basal state that was pair-housed (averse-paired) had a complete change of preference from averse to seeking by decreasing P2 (the best advantageous choice) less than 60% and simultaneously increasing P3 (disadvantageous choice) more than 20% (Fig. 3b, left upper panel). The group with a risk-seeking inclination at the basal state that was pair-housed (seeking-paired) displayed deeper risk-seeking, by further decreasing P2, even close to the score for P3 (Fig. 3b, right upper panel). The most dramatic change toward risk-seeking occurred in the group of rats that had a risk-seeking inclination at the basal state and were housed in isolation (seeking-isolated), as the preferences for P2 and P3 were significantly reversed (i.e., for P2, it approached 20% and for P3, 60%) (Fig. 3b, right lower panel). The results indicate that this group became seriously aggravated by cocaine to a more severe level of risk-seeking behavior. The most interesting group was the one with a risk-averse inclination at the basal state and housed in isolation (averse-isolated), as no change of preference was observed throughout the entire time-course of the cocaine sensitization scheme (Fig. 3b and c, left lower panel). The interaction between the basal risk-averse trait and isolated housing seemed to strongly cause this group to be more likely to maintain their existing risk-averse preference by an unknown mechanism.

Further analysis of several choice-related behavioral parameters^7, 24^ from the data obtained during the cocaine challenge revealed some interesting findings. Remarkably, in all three groups that manifested differential change of preference upon cocaine challenge, only the cocaine, compared to the saline pre-exposed sub-groups, showed significantly higher scores in one of the major parameters, namely reward latency, premature response, and perseverative screen touch (Fig. 4). While the higher scores in premature response and perseverative screen touch may be associated with impulsivity and compulsivity, respectively, as shown in the literature^30^, we speculate that the higher score in reward latency was likely to arise because rats were more interested in something other, such as the screen (or light), than the pellet reward itself. Interestingly, the correlation analysis for these data revealed that there were significant positive correlations between these parameters (except perseverative response) and disadvantageous choices (P3 + P4) (Fig. 5a–c). Although we are not sure whether these parameters conspicuously arisen as a result of change of preference or they contributed to perform the preference change, these results strongly imply that the change of preference observed after the cocaine challenge was accompanied by change in different parameters, depending on the group. Meanwhile, the averse-isolated group, which did not respond to the cocaine challenge, nonetheless showed significant increases in some minor behavioral parameters such as omission, correct response latency, and feed-tray entries in cocaine compared to the saline pre-exposed sub-groups (Fig. 4, left lower panel). Interestingly, it was found that there were positive significant correlations between omission and correct response latency in all, except the seeking-isolated group (Fig. 5d), which may further suggest that only the seeking-isolated group moved beyond a certain point where the rats no longer needed to hesitate to consider which window they would choose in front of the screen, resulting in a loss of association between the two parameters.

In parallel with rGT, we also measured locomotor activity. In the literature, it was previously shown that rats in isolated compared to enriched (or paired) housing respond less to acute cocaine, while they are more likely to develop sensitization^31, 32^. Likewise, when locomotor activity data were analyzed for the paired versus isolated housing groups regardless of traits, we found that the isolated group showed less locomotor activity to acute cocaine, but significantly higher locomotor sensitization than the paired group (Fig. 6b). However, when we examined the individual groups separately, it is very interesting that the averse-isolated group, which showed no change of preference upon the cocaine challenge, was the only group that expressed significant locomotor sensitization (Fig. 6a). Considering that the development of behavioral sensitization is highly associated with drug-seeking behavior^21, 22^, it seems likely that this group shifted their interest to preferably respond to drugs of abuse (i.e., cocaine in the present results) rather than a gambling task. In contrast, rats in the same risk-averse but paired housing group showed a remarkable change toward more risk-seeking preference, with a relatively dull response to locomotor sensitization, suggesting that these rats preferably chose to continuously play with the gambling task with no interest in drugs. The literature suggests that repeated exposure to drugs of abuse or even to gambling causes the dopaminergic pathway to be sensitized to become dysfunctional toward anticipated rewards, rather than actual rewards, which eventually leads to a state of wanting^30, 33, 34^. In rGT, these may be expressed as the increases in reward latency and premature responses in the groups that show preference changes toward risk-seeking behavior (Fig. 4), which are indicative of trying to spend more time on the screen even when actual rewards (i.e., pellets) appeared. Similarly, it is possible that the seeking-paired and seeking-isolated groups also chose to maintain their interest in the gambling task instead of the drugs in response to a sensitized scheme of cocaine, and it may contribute to further reinforce risk-seeking behaviors in rGT without apparent manifestation of locomotor sensitization.

In the present study, we dealt with choice preference in terms of rGT settings with different probabilities of reward and punishment outcomes. In a more general sense, it is worth mentioning that choices involving probabilistic outcomes can be described within a discounting framework, known as probability discounting^35, 36^. As its name implies, individuals have a tendency to discount the value of uncertain outcomes based on the likelihood of their occurrence. Interestingly, it has been reported that probability discounting has been variously affected by repeated administration of psychomotor stimulant drugs. For example, following approximately 3 weeks of withdrawal, repeated amphetamine compared with saline treatment increased risky choice with a decreased probability of receiving the larger reward^37^, while 14 days of cocaine self-administration failed to do so in rats^38^. More recently, cocaine was interestingly shown to dose-dependently decrease the likelihood of condom use in cocaine users when it was associated with the uncertainty for sexually transmitted infection, which is indicative of cocaine’s effect on the reduction of probability discounting^39^. However, although there is some conceptual similarity in probability discounting, rGT moved forward a step further to incorporate the concept of the “risk of losing”, a crucial component of human gambling^13^. Instead of simply “failing to win”, as largely shown in probability discounting task, animals in the rGT experience a “loss” of optimal time to maximize their earnings, similar to monetary loss in human’s IGT^4, 13, 36^. Thus, our present results obtained with the rGT may have better implications for the understanding of pathological gambling in addition to that of general decision-making.

Collectively, our data clearly indicate that a cocaine challenge after chronic pre-exposure to cocaine may become a trigger that aggravates choice preference toward more risk-seeking behavior; however, the quality and level of the change are differentially expressed depending on the interaction between the basal trait (nature) and the housing condition (nurture). Distinct from the situation where locomotor activity is measured alone, as is common in most studies on this subject^20–22^, even cocaine sensitization, when it is combined with the rGT, is differentially expressed depending on the groups, resulting in either sustained increase of locomotor activity (i.e., locomotor sensitization as shown in the averse-isolated group) or being transferred to increase other types of behavior (i.e., an increase in risk-seeking choices as in all other groups). Finally, it largely remains unknown whether our present findings will hold true for age or sex differences, and how the brain affects this process of interaction between different traits and housing conditions with or without cocaine. These questions will certainly lead to more studies on this fascinating topic.

## Methods

### Subjects

Male Sprague-Dawley rats weighing 200–230 g (6 weeks old) on arrival were obtained from Orient Bio Inc. (Seongnam-si, Korea). The rats were first housed three per cage for a 1-week habituation period to a new colony environment, during which they were handled by experimenters and had access to food *ad libitum*. Then, they were separated and kept continuously housed either two (paired) or one (isolated) per cage until the end of the experiments. While being separated, rats were simultaneously placed on a restricted diet with 85% of their normal daily food consumption, which was started 2 days before the pre-training experiments and maintained until the end of the experiments. Food was provided immediately after the daily training session to sufficiently maintain the animals’ growth and motivation. Water was available *ad libitum* at all times. Colony rooms with a controlled room temperature (21 °C) were set for a 12-hour light/dark cycle (lights on at 8:00 am) and all experiments were conducted during the day time. All animal use procedures were conducted according to an approved Institutional Animal Care and Use Committee protocol of Yonsei University College of Medicine.

### Drugs

Cocaine hydrochloride was purchased from Belgopia (Louvain-La-Neuve, Belgium). It was dissolved in sterile 0.9% saline to a final concentration of 15 mg/ml.

### Apparatus

The rat gambling task was conducted in a set of four identical touchscreen-based automated operant chambers housed in dense sound- and light-attenuating boxes (68.6 cm high × 60.7 cm long × 53.5 cm wide) (Campden Instruments Ltd., Leics, UK). Each chamber was equipped with a house light (light-emitting diode), a touch-sensitive liquid crystal display monitor (touchscreen; 15.0 inch, screen resolution 1,024 × 768), a pellet dispenser, and food magazine unit (with light and infrared beam to detect entries) facing opposite the touchscreen. The chambers had a trapezoidal shape (30 cm high × 33 cm long [from screen to magazine] × 25 cm wide for the screen and 13 cm wide for the magazine) (Fig. 1a), which is designed to help focus the animal’s attention to the touchscreen and reward delivery area, i.e., the food magazine^8^. On top of the chamber, a transparent lid was secured to the trapezoidal walls with latches to help keep the animals inside the chambers. The floor was constructed from perforated stainless steel, and a tray for litter was located below the floor. The touchscreen used sensitive optical infrared sensors that allow the screen to reliably detect an animal’s soft touch without pressure. Two additional photobeams, positioned on two side walls (6 cm from the screen and 5 cm from the magazine), detected the movement of an animal in the front or rear. A black plastic mask (36 cm high × 28 cm wide) with five response windows (each window size was 3.0 cm high × 3.0 cm wide, and positioned in a row with each windows spaced 1.0 cm apart, 3.5 cm from the grid floor) was fitted in front of the touchscreen, which helped reduce accidental screen touches and clearly identify the response locations from the background. The visual stimulus with a solid white square of a similar dimension was used as the mask response window, and it was only shown in the left two and right two windows, the middle window was left black. We used the Whisker Standard Software^40^ as the controlling software, which was purchased from Campden Instruments, Ltd., and a single computer was used to control the four chambers.

Locomotor activity was measured with a bank of six activity boxes (35 × 25 × 40 cm) made of translucent Plexiglas. Each box was individually housed in a polyvinyl chloride plastic sound-attenuating cubicle. The floor of each box consisted of 21 stainless steel rods (5 mm in diameter) spaced 1.2 cm apart center-to-center. Two infrared light photobeams (Med Associates, St. Albans, VT, USA), positioned 4.5 cm above the floor and spaced evenly along the longitudinal axis of the box, were used to estimate horizontal locomotor activity. Two additional photo beams, positioned on the sidewall 14.5 cm above the floor and 7.5 cm from the front and back walls, estimated rearing.

### Rat gambling task (rGT): Pre-training

The chambers were kept dark, except when the food magazine, touchscreen, or house light was lit. Animals were trained once daily in a 30 min session, 5 days per week. Each session consisted of a different number of trials within 30 min according to the stage, and each trial started when an animal placed its nose into the magazine. If the designated trials for each stage were completed before 30 min, the session was terminated early. In stage 1, animals were first habituated to the touchscreen chamber for one session, during which 10 reward pellets were placed in the food magazine just to attract the animals’ attention toward the pellets regardless of their action. In this stage, there were no trials, and the screen did not light up. We used sucrose pellets (45 mg) with chocolate flavor (Bio-Serve, Flemington, NJ, USA) as a reward. In stage 2, animals were trained to learn the relationship between the light stimulus on the screen and the reward pellet over two daily sessions; one of the four windows on the screen was randomly lit for 30 sec. Animals were given three pellets if they touched the window lit, or one pellet even if they failed to do so, within 30 sec. In this stage, only 30 trials were allowed for animals to complete the task. In stage 3, animals learned to touch the screen to receive a pellet over three daily sessions; again, one of the four windows was randomly lit, but it stayed lit until animals touched it. One pellet for each window touch was given, and only 20 trials were allowed for animals to complete the task. In this stage, the inter-trial interval (ITI) of the 5 sec rule was first applied so that animals had to wait for 5 sec after pushing their noses into the food magazine to start a new trial. In stage 4, which lasted over 15–18 daily sessions, animals serially learned to touch one of the four windows randomly lit within different stimulus duration times (starting from 60 sec, then serially reduced to 30, 20, and finally 10 sec) to receive one pellet. Animals completed the task either within 100 trials or during 30 minutes, whichever came first. In this stage, they first time learned that they were punished with a time-out (i.e., the white house light was lit for 5 sec) if they touched the screen without waiting during ITI (premature) or if they did not touch the screen within the duration time (omission). They were also punished if they touched other windows which were not lit. In this way, animals increased the actual number of touches on the right window within a designated number of trials or within the time limit, and, so, they became more accurate in terms of selecting the stimulus that delivers the pellet from others that did not. At that time, the subjects were considered to have acquired the task (i.e., with an ITI of 5 sec and a stimulus duration of 10 sec) when the accuracy was greater than 80% and omissions were fewer than 20%.

### Rat gambling task (rGT): rGT-training

The design of the rGT was based on Zeeb *et al*.’s study (2009)^7^, in which the animals were confronted with four choices differing in the probability and magnitude of reward (food) and punishment (time-out), and then they had to learn an optimal strategy to determine which choice provided the most reward earning per session. In stage 5, which lasted over 7 daily sessions, animals learned for the first time the relationship between each window and the different reward/punishment ratio assigned to that window, which was as follows: window (P1), 1 pellet (90%) or 5 sec time-out (10%); window (P2), 2 pellets (80%) or 10 sec time-out (20%); window (P3), 3 pellets (50%) or 30 sec time-out (50%); and window (P4), 4 pellets (40%) or 40 sec time-out (60%). In this stage, one of the four windows was randomly lit for 10 sec as before; there was no trial limit and the animals completed the task just within 30 min; animals were no longer punished either by omission or by touching windows with no light, whereas they were continuously punished (i.e., the white house light was lit for 5 sec) by a premature response. Additionally, for the first time in this stage, animals were punished (time-out; i.e., the white house light was lit, and simultaneously all the windows on the screen flashed for 5 to 40 sec) even by correctly touching the screen according to the pre-designated schedule for each window. So far, from stages 1 to 5, only one of four windows on the screen was randomly lit. However, now, for the first time in stage 6, all four windows were simultaneously lit when each new trial started, and animals were allowed to wait for 5 sec of ITI and then choose one of the four windows, which was lit for 10 sec. The reward and punishment settings designated for each window were the same as introduced in stage 5. Now, depending on which window the subjects chose, they would receive either reward (pellet) or punishment (time-out) with differently programmed probabilities. Once a trial was finished with whatever outcome, they faced again four different choices in the next trial and this process was repeated until 30 min passed. Hypothetically, if one window was exclusively chosen, the amount of reward pellets per session that animal could obtain was as follows: P1, 295; P2, 411; P3, 135; and P4, 99 pellets^41^. The percentage of choices (number of choices for a specific window divided by a total number of choices made × 100) was used to measure the animals’ preferences for the different windows. After 15 daily sessions were completed, the average of the last three daily sessions’ choice percentages was considered as a basal score for the animals’ risk preference. Animals were categorized as risk-averse when the basal score for P2 (the best optimal choice) was equal to or higher than 60%, whereas they were categorized as risk-seeking when it was lower than 60%. To avoid any location bias, windows were allocated in a counterbalanced way as follows: for half of the animals, the windows were 1 (P1), 2 (P4), 3 (P2), and 4 (P3); for the other half of the animals, the windows were 1 (P4), 2 (P1), 3 (P3), and 4 (P2). In addition to the previously introduced premature response and omission (both were expressed as a percentage of the total number of trials initiated), choice-related behavioral parameters such as a perseverative response (repeatedly touching the screen during punishment, calculated as the total number of screen touches divided by the total duration of punishment), feed-tray entry (repeatedly entering the food magazine, calculated as the number of feed-tray entries divided by the number of trials (including omissions) × 100), reward latency (the duration of time that passed for animals to get the reward after a screen touch when it was rewarded), and correct response latency (the duration of time that passed for animals to correctly touch the screen from the end of the ITI while the screen was lit) were analyzed.

### Design and procedures

Experimental schemes are briefly depicted in Fig. 1b. Two days after the rats were separately housed either paired (n = 30) or isolated (n = 36) per cage, they started to be serially trained in stages 1 to 6. Once the rats were categorized as either risk-averse or risk-seeking according to the average score of P2 for the last 3 days of stage 6 training, the rats in each group (i.e., the averse-paired, averse-isolated, seeking-paired, and seeking-isolated groups) were sub-divided into saline pre-exposed or cocaine pre-exposed groups, resulting in eight different sub-groups.

During the pre-exposure phase, rats in different groups were administered with saline or cocaine (15 mg/kg, intraperitoneally [i.p.]), once daily for 7 days. Rats were placed in the locomotor activity boxes for 60 min only after their first (day 1) and last (day 7) injections, whereas the remaining injections (days 2~6) were given in the home cage without measuring locomotor activity. This procedure of injecting cocaine is commonly used to develop sensitization to cocaine^25, 26^. After 2 weeks of a drug-free withdrawal period, rats were again placed in the activity boxes and first allowed to habituate for 30 min before testing for sensitization. Then, they were all administered with a challenge injection of cocaine (15 mg/kg, i.p.), and they were immediately returned to the activity boxes where activity counts were measured for 60 min. Thirty minute rGT sessions were conducted immediately following locomotor activity measurements (pre-exposure days 1 and 7, and the challenge day) or directly from the home cage (the last 5 days of the withdrawal period). After the data were obtained during the challenge day, 7 rats (2 from paired and 5 from isolated) with fewer than five trial numbers were excluded from all further analysis, and ultimately 59 rats (28 in paired and 31 in isolated) were used in the data analysis presented herein.

### Statistical analysis

Data are shown as mean + standard error of the mean, and they were analyzed using Sigma Plot, version 12.5 (Systat Software, Inc., Chicago, IL, USA). An arcsine transformation was performed for the data obtained as the percentage before the analysis. The analysis of choice preference for the four windows (P1~P4) between the basal and challenge states was performed using two-way repeated-measures analysis of variance (ANOVA) with the basal or challenge states as the within-subjects factor and choice (P1~P4) as the between-subjects factor. Analysis of the time-course effects between saline and cocaine pre-exposures for P2 or P3 was performed using two-way repeated-measures ANOVA with the time-course as the within-subjects factor and the different pre-exposures as the between-subjects factor. Data from the locomotor activity combined with paired/isolated and averse/seeking were analyzed using a two-way ANOVA with the pre-exposures as the within-subjects factor and paired/isolated or averse/seeking as the between-subjects factor. Post-hoc analysis was performed using Bonferroni comparisons. Analysis of the choice-related behavioral parameters (reward latency, premature response, omission, perseverative response, correct response latency, feed-tray entry, and locomotor activity) between saline and cocaine pre-exposures on the challenge day was performed using the independent sample t-test. Possible correlations between the behavioral parameters (reward latency, premature, and perseverative response) and P3 + P4 were examined using the Spearman rank-order correlation. Differences between experimental conditions were considered statistically significant when *p* < 0.05.
